# Medullary Carcinoma of the Colon: Not as Favourable as Previously Thought?

**DOI:** 10.1007/s12029-025-01304-x

**Published:** 2025-09-11

**Authors:** Daniel Netto, Mugtaba Dafalla, Karim Muhammad, Haithum Tumeh, Chloe Sowerby, Konstantinos Kamposioras

**Affiliations:** 1 Department of Medical Oncology, The Christie NHS Foundation Trust, Manchester, UK; 2 Department of Pathology, Stockport NHS Foundation Trust, Stockport, UK; 3 Department of Surgery Tameside and Glossop Integrated Care NHS Foundation Trust, Ashton-under-Lyne, UK; 4 Division of Cancer Sciences, School of Medical Sciences, Faculty of Biology, Medicine and Health, Manchester Academic Health Science Centre (MAHSC), Manchester Cancer Research Centre, University of Manchester, 555 Wilmslow Road, Manchester, M20 4GJ UK

**Keywords:** Medullary carcinoma of the colon, Mismatch repair-deficient phenotype, Immunochemical test

## Abstract

**Introduction:**

Medullary carcinoma of the colon (MCC) is a rare, evolving subtype of colorectal cancer that is histologically and clinically distinct from poorly differentiated and undifferentiated adenocarcinoma. Comprehensive meta-analyses show that MCCs predominantly have a mismatch repair-deficient (MMRd) phenotype, which typically predicts a more favourable prognosis.

**Case Presentation:**

We present a unique case involving a male patient with an aggressive presentation of colon cancer. Histopathology supported a diagnosis of MCC, initially staged as T3N0M0. Shortly after resection, the patient presented with rapidly progressing metastatic disease involving brain and subcutaneous metastases and subsequent mortality.

**Conclusion:**

MCC is an understudied subpopulation of colon cancers. Historically, the presence of MMRd in early-stage colon cancer predicts a favourable prognosis and also a lack of benefit from adjuvant chemotherapy. Whether this also extrapolates to MCCs remains unclear, and as such, the case presented highlights the uncertainty in the current evidence base. Further research is warranted to help inform robust clinical management.

## Introduction

Medullary carcinoma of the colon (MCC) is a rare entity, constituting ~ 0.3% of all colorectal cancers [1]. MCC was recognised by the World Health Organisation in 2010 as a distinct histological subtype of colorectal cancer that historically was synonymised under the spectrum of poorly or undifferentiated adenocarcinomas. With greater recognition, MCCs are now thought to comprise ~ 20% of all large bowel poorly differentiated adenocarcinomas (PDAs) [2]. This distinction is clinically important as recent comparative studies suggest that MCCs carry a more favourable prognosis, with median overall survival for MCC 82.0 months compared with 43.9 months for PDAs and 47.3 months for undifferentiated carcinomas (UDCs) [3, 4].

Demographics from meta-analyses suggest a higher prevalence of MCCs in the female sex and in patients aged > 70 years [2, 3]. MCCs exhibit a clear pattern favouring right-sided colon presentation, predominantly affecting the caecum and ascending colon, with a higher T stage [2, 5]. Further retrospective analyses have demonstrated that MCCs tend to mostly present at stage II with infrequent occurrences of synchronous lymph node or distant metastasis comparative to adenocarcinomas [2, 3, 5].

Histopathological analysis is critical to the diagnosis. Morphologically, MCCs are characteristically arranged into sheets of malignant cells with minimal to no glandular differentiation, small- to medium-sized vesicular nuclei, a large prominent nucleolus, and eosinophilic cytoplasm [6, 7]. The presence of microsatellite instability is at a much higher frequency than in PDAs and UDCs [8]. The tumour immune microenvironment in MCCs also appears distinctly different with an observed dense lymphocytic infiltration [2, 7]. One study has shown specific enrichment of CD8 +, PD-1 +, PD-L1 +, and Foxp3 + lymphocyte counts in the tumour and surrounding stroma, even compared to other MSI-H colorectal cancers [8]. Upregulation of IFN-γ gene signalling pathways has also been observed; IFN-γ itself was upregulated with subsequent downstream amplification leading to a 4.29 fold increase in *IDO-1* expression [8, 9]. *IDO-1* is implicated in the promotion of tumour directed immune tolerance and subsequent immune evasion. Similarly, the upregulation of PD-1 and PD-L1 may also be implicated in tumourigenesis [8]. Furthermore, there appears to be a lower rate of rat sarcoma virus (*RAS)* mutations and higher rates of *BRAF *(V600E) mutations in MCCs [2, 4].

It is difficult to differentiate between MCC and PDC using light microscopy alone, as both tumours lack glandular structures. Therefore, immunohistochemical stains are helpful in distinguishing between these two entities [10]. Winn et al. and Lin et al. have looked at a wide range of immunohistochemical stains to differentiate MCC from PDC [11, 12]. Approximately 73% of MCCs have showed positive staining for calretinin compared to 12% of PDCs. CDX-2 was positive in 19% of MCCs compared to 55% in PDCs [11]. These tumours frequently retained positive staining for MUC-1, MUC-2, SATB2 and TFF-3, suggesting that these tumours maintain some degree of intestinal differentiation [11, 12].

The QUASAR trial demonstrated that microsatellite instability (MSI-H) was a positive prognostic factor in colorectal cancers [13]. Furthermore, the emergence of the Immunoscore has strengthened dogma that tumours with dense lymphocytic infiltration, regardless of MSI status, confer better recurrence-free survival and overall survival [14, 15]. These factors, which are commonly observed in MCCs, may explain the favourable prognosis that is conferred [16]. Clinicians may therefore deduce from literature that MCC is considered a prognostically favourable histological subtype. However, we present a case below that challenges this belief.

## Case Presentation

A 70-year-old male patient presented to primary care in October 2023 with a history of weight loss, altered bowel habits, and abdominal pain. Following a positive faecal immunochemical test, he was referred for urgent colonoscopy under the 2-week waiting pathway. His medical history included chronic obstructive pulmonary disease, stage 3 chronic kidney disease, hypothyroidism of unknown aetiology, psoriasis, and intermittent claudication of the lower limbs. His sister had passed away from colorectal cancer in her mid-60s, but we were unaware of the details of her histology or treatment. At presentation, he had an Eastern Cooperative Oncology Group (ECOG) performance status of 1.

A colonoscopy was performed 2 weeks later and revealed a malignant polypoidal mass measuring 45 mm extending from the proximal ascending colon to the cecum. Biopsies were taken and sent for histological evaluation, which showed poorly differentiated malignant cells with weak patchy staining for CDX2, AE1/3 and negative for LCA and HMB45. CT staging was performed as part of the pre-operative work-up and showed a larger 9 cm tumour extending from the proximal ascending colon to the level of the ileo-caecal valve, with the appearance suggesting early extramural extension. A few small indeterminate local lymph nodes were noted, but no distant disease; therefore, the clinical stage was T4a N1M0.

Seven weeks after the initial colonoscopy, the patient underwent a right hemicolectomy.

**Fig. 1:**
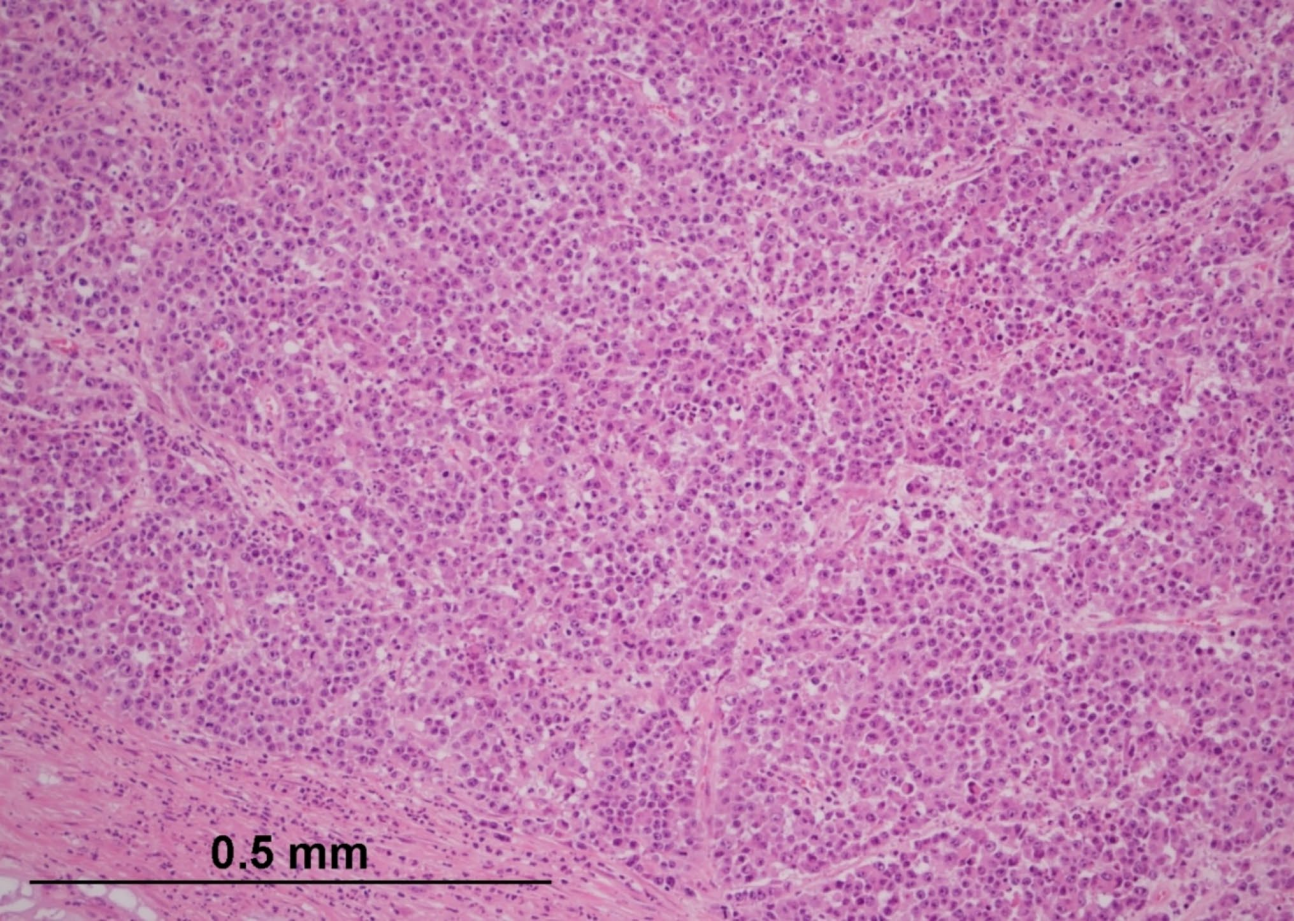
Medullary carcinoma of the colon showing a solid growth pattern. The tumour forms nests and sheets of malignant cells infiltrating the colonic wall. Haematoxylin and eosin (H&E, × 100) (courtesy of Dr Michael Scott, Manchester University NHS Foundation Trust)

**Fig. 2:**
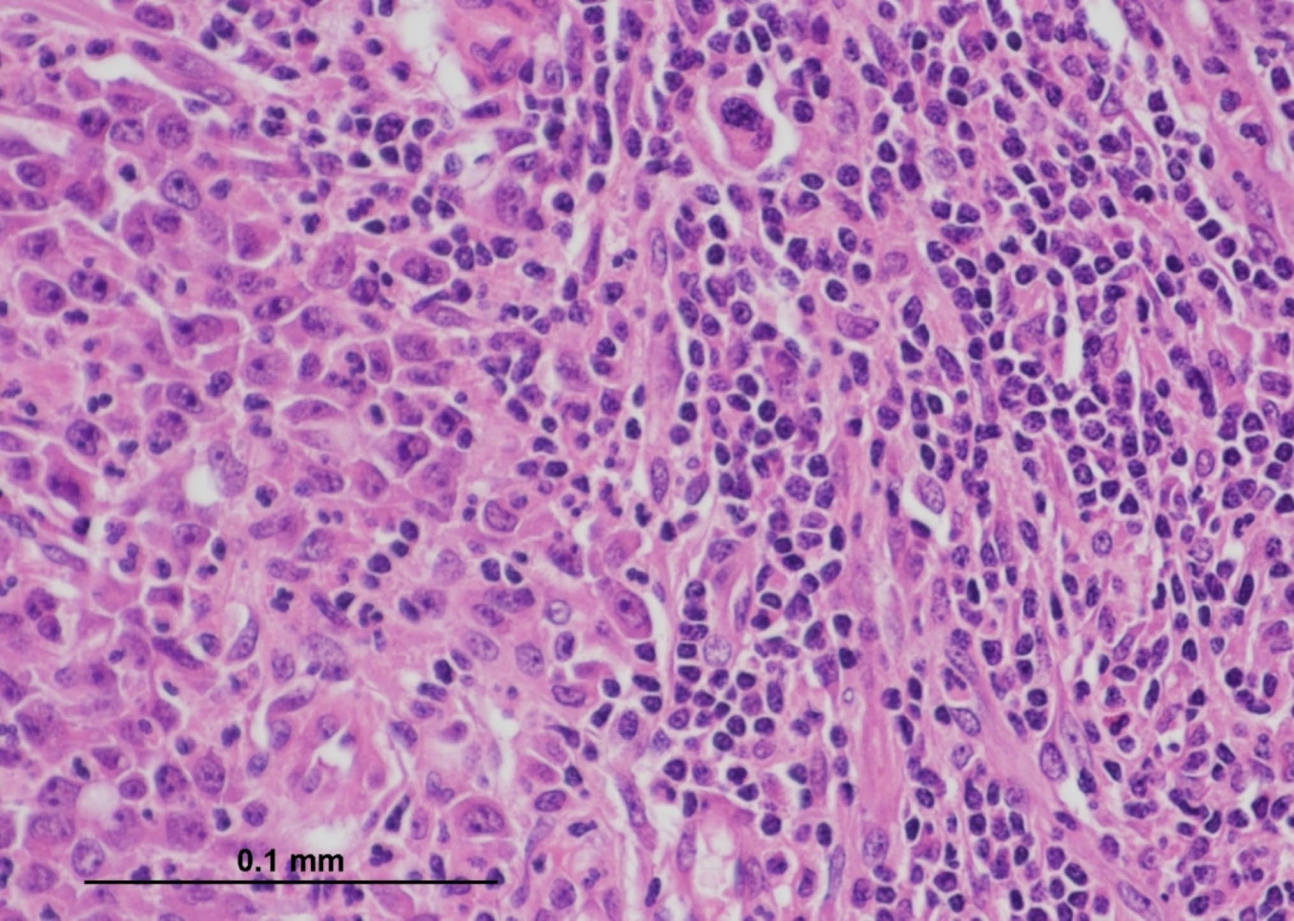
High-power view of the tumour highlighting large tumour cells with vesicular nuclei, prominent nucleoli, and abundant eosinophilic cytoplasm. Numerous intra-tumoural and stromal lymphocytes are present (H&E, × 400) (courtesy of Dr Michael Scott, Manchester University NHS Foundation Trust)

**Fig. 3:**
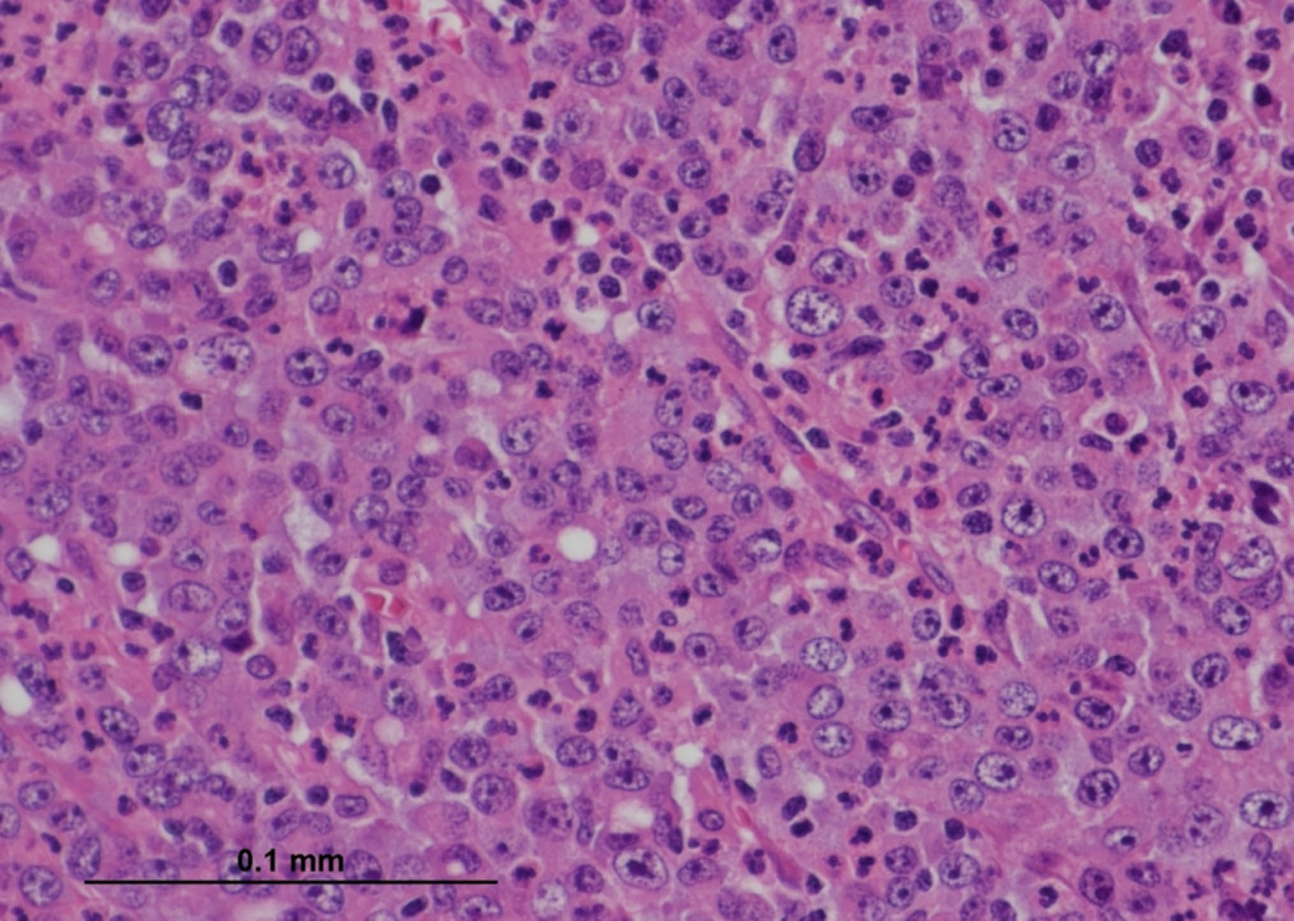
Histological section showing regions of the tumour with a prominent intratumoural neutrophilic infiltrate exceeding lymphocytic infiltration (H&E, × 400) (courtesy of Dr Michael Scott, Manchester University NHS Foundation Trust)

Histological examination of the primary tumour revealed a poorly differentiated malignant neoplasm with a solid growth pattern (Fig. 1). The tumour cells contained a moderate amount of amphophilic and eosinophilic cytoplasm, with pleomorphic, vesicular nuclei and prominent nucleoli. In addition to widespread necrosis, intra-tumoural lymphocytes and neutrophils were identified, albeit in a patchy distribution, with some areas showing a predominance of neutrophils over lymphocytes (Figs. 2 and 3). Extramural lymphatic invasion, perineural invasion, and lymphovascular invasion (LVI) were present, constituting high-risk pathological features. All 21 identified lymph nodes were negative. The pathological staging was therefore pT3 pN0, with clear surgical margins (R0 resection).

**Fig. 4:**
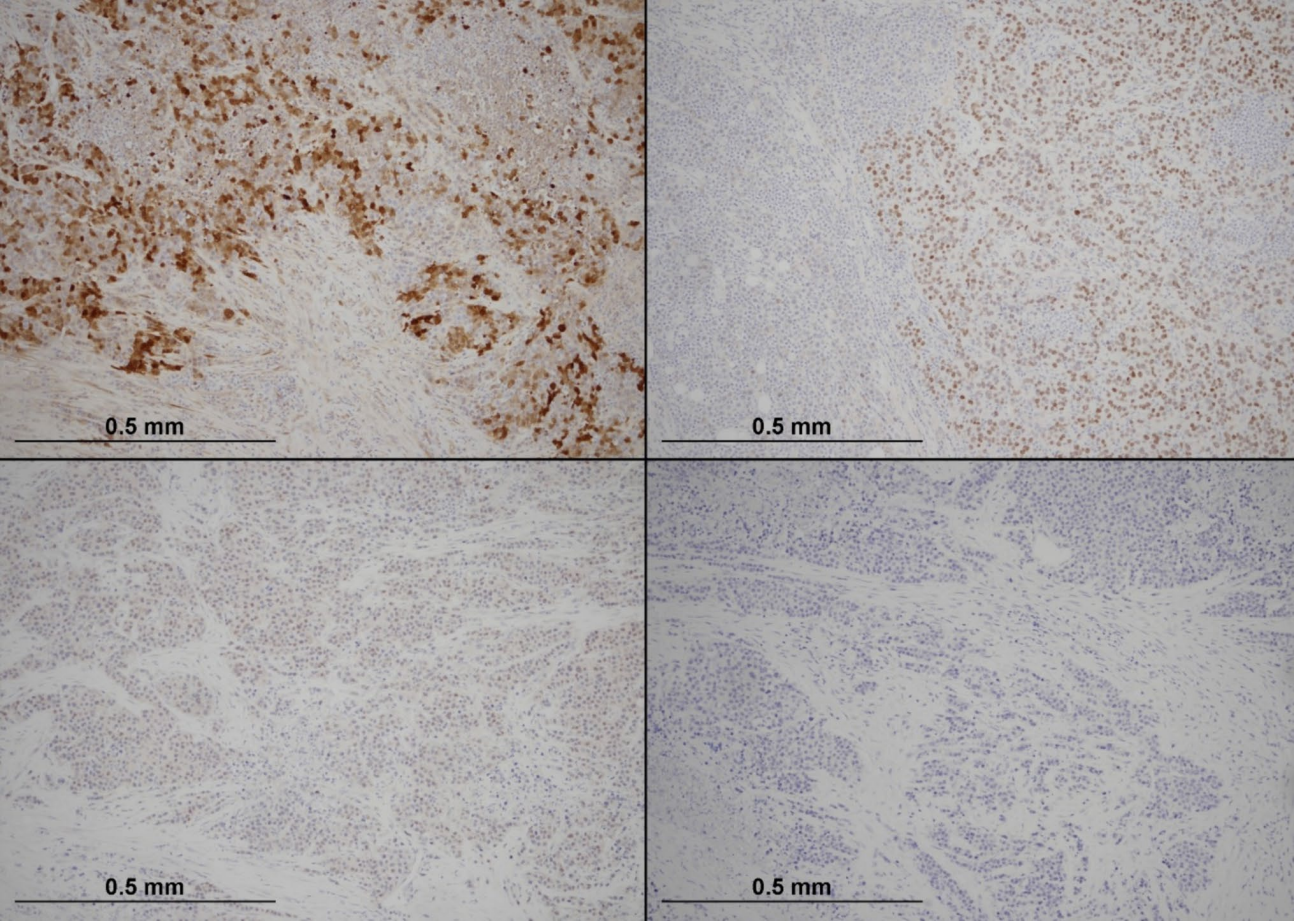
Immunohistochemical staining profile of the tumour (× 100). The top left picture shows diffuse nuclear and cytoplasmic positivity for calretinin. The top right demonstrates patchy nuclear staining for SATB2. The bottom left shows focal CDX2 nuclear positivity, whilst the bottom right reveals complete absence of CK20 expression

Immunohistochemistry stains were performed. The tumour cells showed positive staining for calretinin (Ventana; clone SP65, RTU), with very focal positive staining for SATB2 (Master Diagnostica; clone EP281, RTU). There was weak focal staining for CDX2 (Ventana; clone EPR2764Y, RTU), whilst CK7 (Ventana; clone SP52, RTU) and CK20 (Ventana; clone SP33, RTU) were negative (Fig. 4). The tumour cells were also negative for chromogranin-A (Ventana; clone LK2H10, RTU). There was focal positive staining for synaptophysin (Ventana; clone SP11, RTU).

**Fig. 5:**
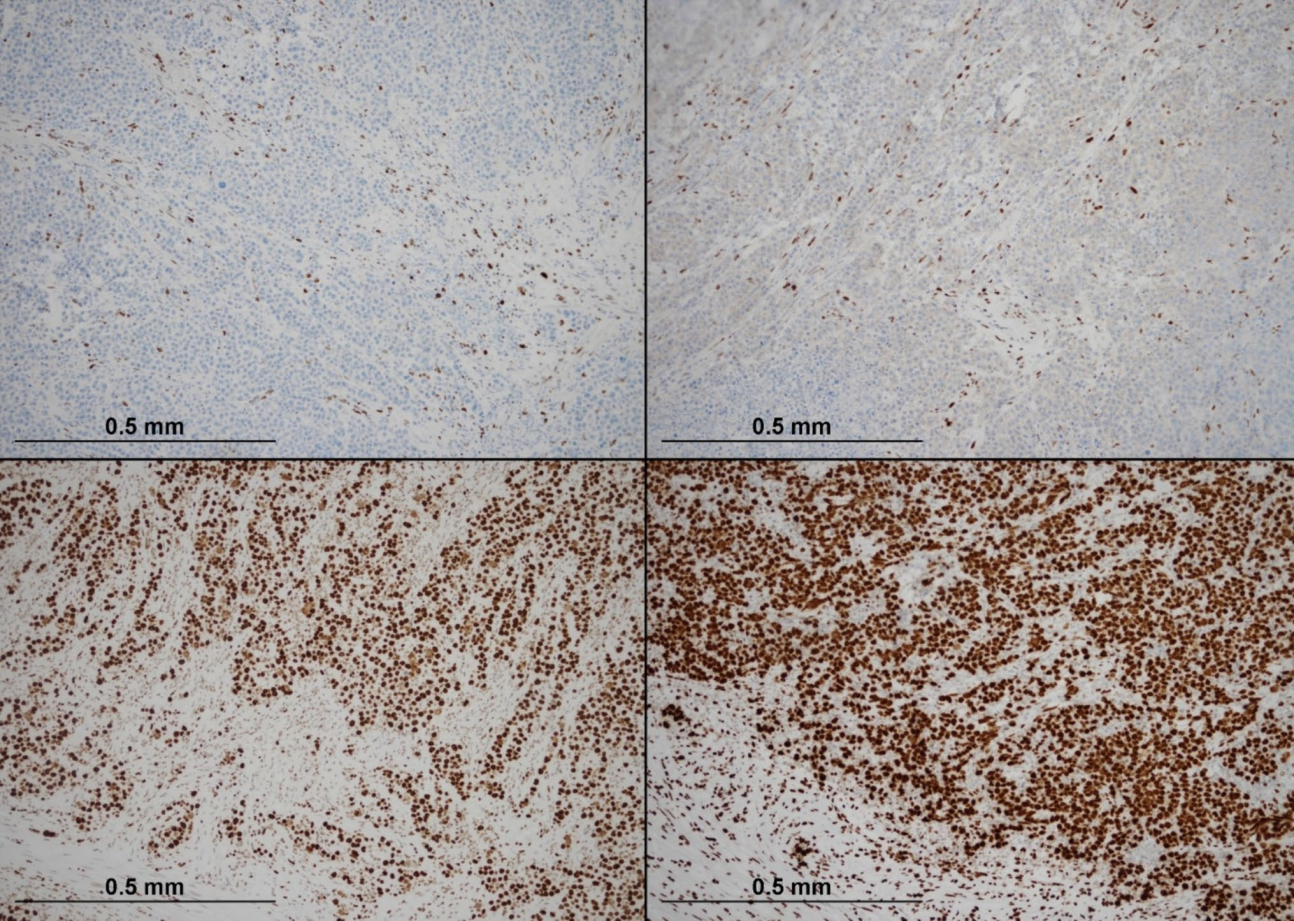
Mismatch repair (MMR) protein immunohistochemistry in the tumour (× 100). The top left quadrant shows complete loss of nuclear MLH1 expression in tumour cells, with intact staining in background stromal and inflammatory cells. The top right quadrant demonstrates concurrent loss of PMS2. In contrast, the bottom left and bottom right quadrants show retained nuclear expression of MSH2 and MSH6, respectively, nucleoli (courtesy of Dr Michael Scott, Manchester University NHS Foundation Trust)

Mismatch repair (MMR) protein analysis showed loss of MLH1 and PMS2 expression, with retention of MSH2 and MSH6 (Fig. 5). MMR immunohistochemistry was performed at Manchester University NHS Foundation Trust. MLH1 promoter hypermethylation was detected (41%), and a BRAF V600E mutation was identified. Both MLH1 and BRAF testing were carried out at the North West Genomic Laboratory Hub, a UKAS-accredited medical laboratory (no. 9865).

KRAS and PD-L1 status were not assessed, and Immunoscore analysis was not performed as this is not current standard practice.

The overall constellation of morphological and immunohistochemical features supported a diagnosis of MCC arising sporadically.

The patient was referred to medical oncology 4 weeks later to discuss the benefit of adjuvant chemotherapy. The case was reviewed in the context of stage II disease, PNI, LVI, and MMRd; the pre-planned consensus from our oncology service was to not offer adjuvant chemotherapy, as it would typically offer minimal survival benefit for MMRd tumours [17]. The review was conducted by telephone, as the patient was too unwell to attend in person. He reported a subcutaneous lump in the groin that had grown to 3 cm in diameter over the previous 2 weeks. He also described symptoms of bilious vomiting, severe headaches, and ataxia. Urgent medical assessment at his local accident and emergency department was recommended, where he was admitted.

An MRI brain scan was performed and showed a solitary right cerebellar metastasis with significant oedema and mass effect on the fourth ventricle. He underwent an extracranial restaging CT scan which unfortunately showed a new liver lesion of 1.3 cm, a subcutaneous lesion in the left iliac fossa of 2.3 cm and left inguinal lymphadenopathy, confirming the clinical suspicion of metastatic disease. His case was discussed with neuro-oncology, and given the potential for immunotherapy, he was offered brain metastectomy. He underwent surgery on the 1 st March 2024; the cerebellar metastasis had a morphological appearance similar to a primary colonic metastasis (Fig. 4).

**Fig. 6:**
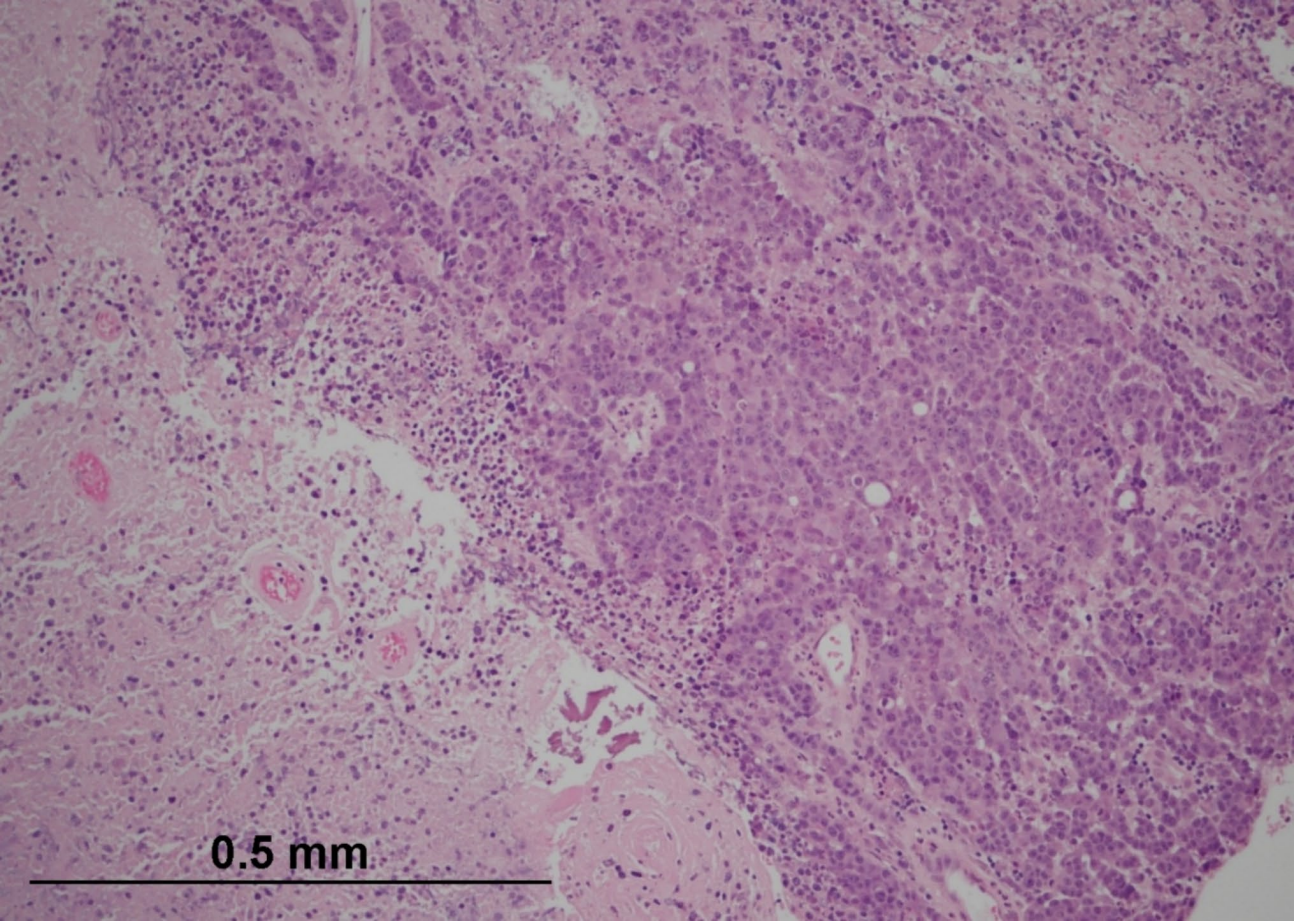
Histological section of brain metastasis (H&E, × 100). The tumour shows similar morphology to the previously described colonic tumour (courtesy of Prof Federico Roncaroli, Salford Royal NHS Trust)

**Fig. 7:**
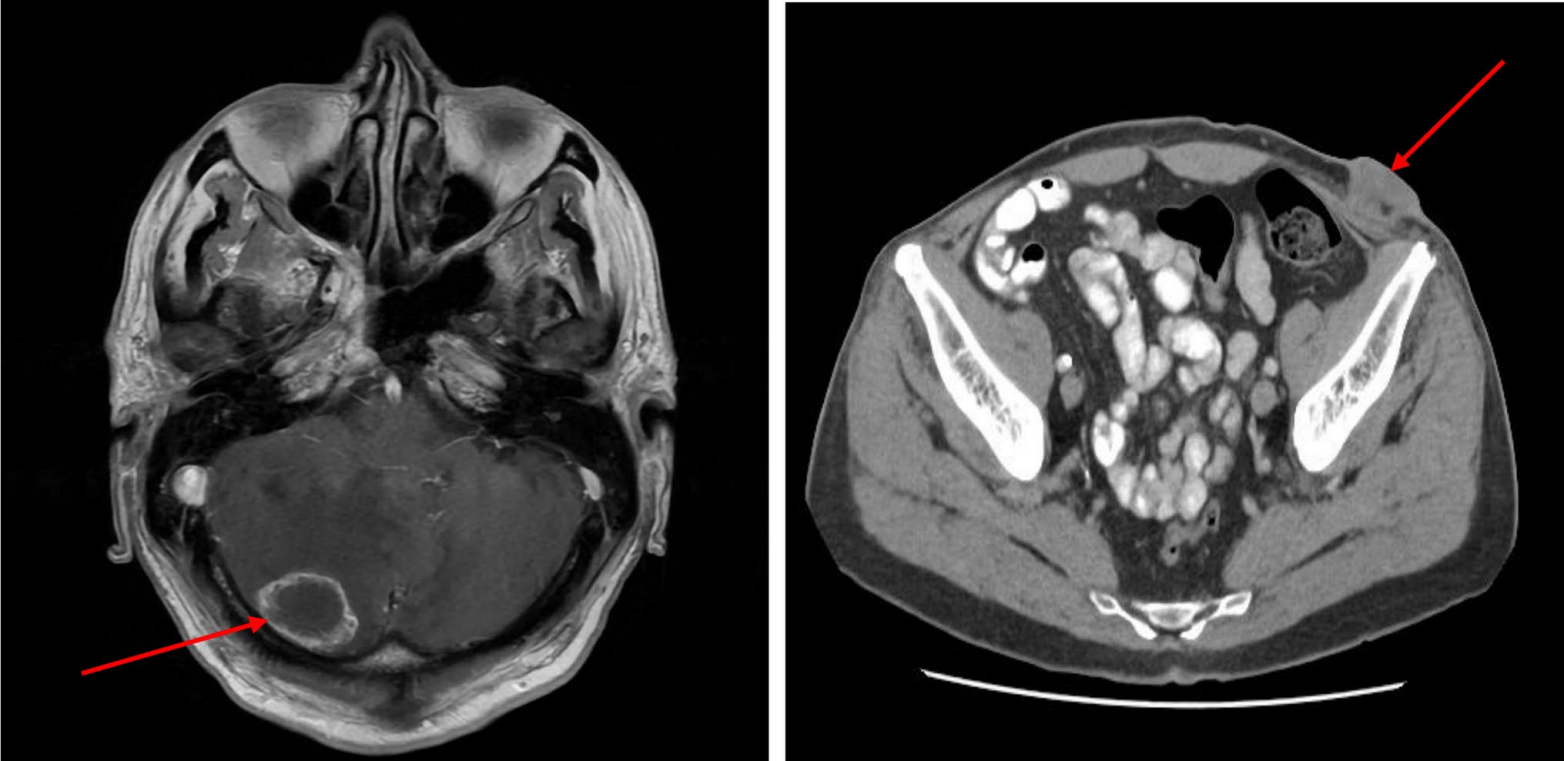
First post-operative staging showing new solitary right cerebellar metastasis (right) and subcutaneous metastasis (left)

**Fig. 8:**
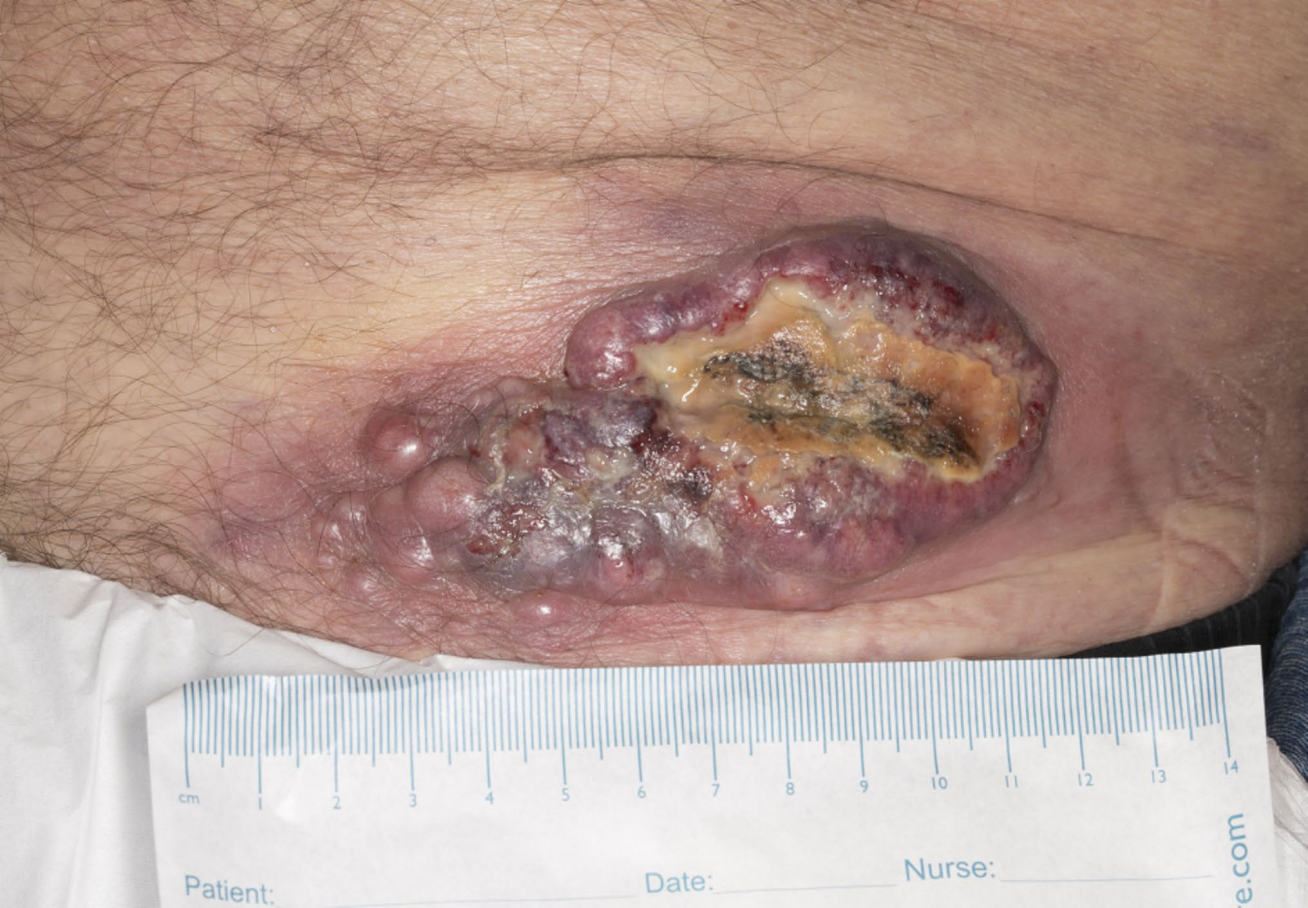
Medical photography from 18th March 2024 showing enlarging and fungating subcutaneous metastasis. The patient was consented for pembrolizumab

A multidisciplinary decision was made to delay post-operative SRS in the resection bed in favour of accelerating the start of pembrolizumab 3 weeks later. Chemotherapy was considered as an alternative, but there were concerns about potential toxicity given his deteriorating performance status. He was reviewed face-to-face in the medical oncology clinic, where concerns about his increasing frailty were discussed, but it was ultimately decided to attempt to start pembrolizumab after careful risk-benefit discussions. Unfortunately, shortly after starting the first cycle of systemic treatment, he was readmitted to hospital with confusion and abdominal pain. A CT scan during hospitalisation confirmed significant progressive disease with enlarging hepatic, subcutaneous, and lytic bone metastasis in addition to new lung lobar consolidation. Despite all supportive measures, including parenteral nutrition and antibiotics, the patient eventually died 25 days after treatment initiation (Figs. 6, 7, and 8).

## Discussion

Clinicopathological features in this patient were consistent with a diagnosis of MCC, including age (70 years), location (ascending colon), large size (9 cm), LVI, MMRd, and BRAF V600E status. Conversely, male sex and presence of PNI are less frequently expected [4, 7].

PNI appears to only have a modest impact on overall survival (HR 1.18), and male sex is not identified as a prognostic factor [3, 4]. Therefore, these characteristics do not explain the aggressive course of this patient’s cancer. It is likely that the presence of LVI is a pertinent risk factor for relapse (HR 1.49) and is recognised as such in guidelines of stage II adenocarcinoma when deciding upon suitability for adjuvant chemotherapy [4]. Tumour size > 7 cm may also be an independent risk factor in MCC (HR 1.66) and chemotherapy may be beneficial (HR 0.73) [3]. Despite this, it is also well established that MMRd is a strong positive prognostic predictor in localised colon cancer and that stage II patients do not benefit from adjuvant chemotherapy [18]. The current ASCO and ESMO guidelines therefore do not recommend administering adjuvant chemotherapy to patients with stage II MMRd colon cancer. Whether this conclusion can extrapolate to MCCs, an understudied subpopulation, remains unclear and further evidence is required.

There is limited data on MCC to corroborate our experience. The difficulty in diagnosis means that prevalence is likely underreported. One case report described a patient who developed metastatic disease in the peri-oesophageal lymph nodes shortly after surgical resection for presumed stage III ascending colon cancer. The patient subsequently progressed despite chemotherapy and died a few months after her initial diagnosis [19]. A retrospective single-centre analysis of 33 MCC patients showed inferior overall survival compared with the PDA/UDA cohort, but there were significant confounding differences between the two populations, including higher frailty scores and lower rates of adjuvant chemotherapy in the MCC group, which may partially explain the differences in survival [20]. This may provide a signal that patients with MCC subtype benefit more from adjuvant chemotherapy than we would normally anticipate. Another single centre case series study looked at 10 cases of MCC in comparison to 22 cases of PDCs with MSI-H disease. In the MCC cohort, 70% presented with stage III disease and 60% received adjuvant chemotherapy. Comparatively, in the PDA group, 96% of patients presented with stage III disease and 86.4% received adjuvant chemotherapy. Interestingly, 5-year OS was 42.9% vs 76.6% (MCC vs PDA group, respectively) despite more patients in the MCC group presenting at an earlier stage. Univariate analysis identified MCC as a poor prognostic factor. A significantly lower proportion of patients in the MCC group received adjuvant chemotherapy, and therefore, an alternative explanation is that the benefit of adjuvant chemotherapy for MCCs may be higher than typically anticipated in MSI-H disease [21]. In addition, there is increasing evidence to challenge the dogma that MSI high status correlates strongly with a favourable prognosis, particularly in cases with the presence of metastatic disease [22, 23].

When the patient developed metastatic disease, treatment with pembrolizumab was offered in accordance with well-established data from KEYNOTE 177, which demonstrated a significant benefit for patients with MMRd/MSI-H metastatic colorectal cancer. Whilst it is plausible that we were simply unable to deliver enough treatment to achieve meaningful benefit, it should be noted that MCCs are unlikely to have been represented in the trial population [24]. Furthermore, due to constraints of patient performance status, we were unable to safely consider fluorouracil-based chemotherapy. Patients presenting with de novo stage IV disease are thought to have a relatively poor prognosis with median OS of only 10 months [3]. There is consensus that patients with *BRAF* mutations may respond less well to chemotherapy and represents an independent risk factor in early-stage disease [25]. This notable point may also adversely affect the survival of patients with MCC in both early and advanced stage disease.

According to the current literature, including large database analyses, MCCs appear to follow a more indolent disease course compared to PDA and UDCs, presenting less frequently with de novo metastasis and with better overall survival. Our case contradicts this notion, as the patient presented with stage II disease, but within 3 months of diagnosis developed recurrent disease with brain, liver, and rapidly progressing subcutaneous metastases. To our knowledge, this is the first case of MCC presenting with this accelerated trajectory of disease.

## Conclusion

MCC is an understudied disease with a low incidence, resulting in a limited understanding of its clinical presentation. Resultantly, clinicians should recognise the limitations of retrospective data and case series to avoid drawing definitive conclusions as prognosis may be overestimated. Critical questions remain as to how classical high-risk factors such as PNI and LVI may apply in cases of MCC; we postulate that greater emphasis should be placed on these factors compared to poorly differentiated and undifferentiated MMRd colon cancers as they may carry a higher prognostic importance in MCCs. The case also highlights the pitfalls and current limitations of prognostic classifications such as TNM and Immunoscore, considering that our patient presented with stage II disease and, if performed, would have likely had a high Immunoscore, both of which are associated with improved survival.

This case prompts clinicians to consider MCC as a distinct entity from an adenocarcinoma with MMRd. Further molecular characterisation and clinical data analysis of MCCs is required to identify other pertinent prognostic parameters and predictive biomarkers of treatment response and survival outcomes.

## Data Availability

No datasets were generated or analysed during the current study.
